# Morphological characterization and phylogenetic relationships of Indochinese box turtles—The *Cuora galbinifrons* complex

**DOI:** 10.1002/ece3.5680

**Published:** 2019-10-02

**Authors:** Xiaoli Liu, Wei Li, Zhaoyang Ye, Yanyu Zhu, Xiaoyou Hong, Xinping Zhu

**Affiliations:** ^1^ Key Laboratory of Aquatic Genomics Ministry of Agriculture Key Laboratory of Tropical & Subtropical Fishery Resource Application & Cultivation of Ministry of Agriculture Pearl River Fisheries Research Institute Chinese Academy of Fishery Sciences Guangzhou China; ^2^ College of Life Science and Fisheries Shanghai Ocean University Shanghai China

**Keywords:** *Cuora*, molecular marker, morphology, phylogenetics, turtle

## Abstract

The members of the Indochinese box turtle complex, namely *Cuora galbinifrons*, *Cuora bourreti*, and *Cuora picturata*, rank the most critically endangered turtle species on earth after more than three decades of over‐harvesting for food, traditional Chinese medicine, and pet markets. Despite advances in molecular biology, species boundaries and phylogenetic relationships, the status of the *C*. *galbinifrons* complex remains unresolved due to the small number of specimens observed and collected in the field. In this study, we present analyses of morphologic characters as well as mitochondrial and nuclear DNA data to reconstruct the species boundaries and systematic relationships within the *C*. *galbinifrons* complex. Based on principal component analysis (PCA) and statistical analysis, we found that phenotypic traits partially overlapped among *galbinifrons*, *bourreti*, and *picturata*, and that *galbinifrons* and *bourreti* might be only subspecifically distinct. Moreover, we used the mitochondrial genome, *COI*, and nuclear gene *Rag1* under the maximum likelihood criteria and Bayesian inference criteria to elucidate whether *C. galbinifrons* could be divided into three separate species or subspecies. We found strong support for a sister relationship between *picturata* and the other two species, and consequently, we recommend maintaining *picturata* as a full species, and classifying *bourreti* and *galbinifrons* as subspecies of *C. galbinifrons*. These findings provide evidence for a better understanding of the evolutionary histories of these critically endangered turtles.

## Graphical abstract

Phenotypic traits partially overlapped among *galbinifrons*, *bourreti*, and *picturata*, and *galbinifrons* and *bourreti* might be only subspecifically distinct. We found strong support for a sister relationship between *picturata* and the other two species, and consequently, we recommend maintaining *picturata* as full species, and classifying *bourreti* and *galbinifrons* as subspecies of the *C*. *galbinifrons*.

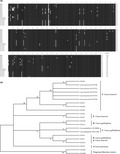

## 1 INTRODUCTION

Turtles have been determined among the oldest known reptiles on earth since the rise of the dinosaurs based on the discovery of the earliest fossil which was found from the Triassic in China (Joyce & Gauthier, 2004). During the last three decades, the excessive exploitation of turtles for the pet trade throughout Asia and, the effects of habitat loss, invasive species, and unclear phylogenetic relationships, combined with the traditional Chinese markets for medicinal and edible purposes, have placed turtles in the center of highly endangered taxa (Gong et al., 2009; Parham, Simison, Kozak, Feldman, & Shi, 2001). According to reports, 51.9% of existing turtle species are recognized as threatened, 20% as critically endangered, and 35.3% are critically endangered or endangered by the World Conservation Union (IUCN TFTSG, 2018). The genus *Cuora*, a member of the Geoemydidae family, are restricted to southeast and east Asia (Takahashi, Ishido, & Hirayama, 2013). Among them in the genus *Cuora*, 12 species are critically endangered and one species is endangered on the IUCN Red List of Threatened Species (IUCN TFTSG, 2018). Given their plight, knowledge of the distribution patterns and the current diversity and phylogeny have significant consequences for species protection (IUCN TFTSG, 2018).

The recently increased data sampling has greatly improved our understanding of the evolutionary relationships of endangered species (Øivind et al., 2011). However, species boundaries, the phylogeny of the genus, and the genetic diversity of populations are still confused and uncertain, as most of the recent records are based on hunters or pet dealers (Parham et al., 2001). For instance, within the *Cuora galbinifrons* complex, varying numbers of recognized species (1–3) have been proposed by separate authors (Fritz & Havaš, 2007; Spinks, Thomson, & Shaffer, 2009; Zhang, Nie, Cao, & Zhang, 2008). While they were initially described as subspecies of *C*. *galbinifrons* (Bourret, 1939), *C. g. bourreti* by Obst and Reimann (1994), and *C. g. picturata* (Lehr, Fritz, Obst, 1998), most recent works follow the recommendation by Stuart and Parham (2004) to treat *Cuora galbinifrons*, *bourreti*, and *picturata* as full species because they are morphologically distinguishable and reciprocally monophyletic and have three major mitochondrial DNA clades (Fritz, Petzold, & Auer, 2006; Stuart & Parham, 2004). However, possible introgression between *bourreti* and *galbinifrons* within the *Cuora galbinifrons* population on Hainan and areas of intergradation in central Vietnam (Fritz & Mendau, 2002), which qualifies *bourreti* and *galbinifrons* as conspecifics under the biological species concept, challenged this conclusion (Fritz et al., 2006). The claim that Hainan hosts possible hybrids as proposed by De Bruin and Artner (1999) has never been substantiated and it is very likely that *bourreti* on Hainan are only from pet trade (Blanck, 2013; Stuart & Parham, 2004); however, the population from Hannan has been described as *Cuora flavomarginata hainanensis* by Li (1958), later included into the synonymy of *C. galbinifrons* by Zhao and Adler (1993) followed by most other authors including Stuart and Parham (2004).

The indefinite geographical range of the wild population, the misinterpretations of molecular‐based phylogenies by different mitochondrial genes or nuclear mitochondrial pseudogenes, and the incomplete lineage sorting and introgression are the main reasons for the ambiguity of taxonomy and systematics of *Cuora* species. In this study, we used nuclear (*Rag1*) and mitochondrial markers (genome and *COI*) in conjunction with morphologic characters to construct the phylogeny among the *C. galbinifrons* complex (*galbinifrons*, *bourreti*, and *picturata*), as these geometric morphometrics and molecular markers have been extensively utilized to investigate the origin and evolutionary history of vertebrates, including the whale shark (*Rhincodon typus*; Md Tauqeer, Petit, Read, & Dove, 2014), Chinese three‐striped box turtle (*Cuora trifasciata*; Li, Zhang, Zhao, Shi, & Zhu, 2015), *Carassius* species complex (Liu, Li, et al., 2017), *Conorhynchos* (Sullivan, Lundberg, & Hardman, 2006), *Hemiculter leucisculus* (Cheng et al., 2018), and Magadi tilapia (Kavembe, Kautt, Machado‐Schiaffino, & Meyer, 2016). We detected morphological differences among the *C. galbinifrons* complex, and moreover, based on the results of nuclear and mitochondrial markers, we investigated whether the *galbinifrons*, *bourreti*, and *picturata* groups can be regarded as separate species or subspecies. The findings of this study further explore the phylogeny of highly threatened *Cuora* and provide significant referenced values for species conservation.

## 2 MATERIALS AND METHODS

### 2.1 Turtle collection and morphometric measurements

**Figure 1:**
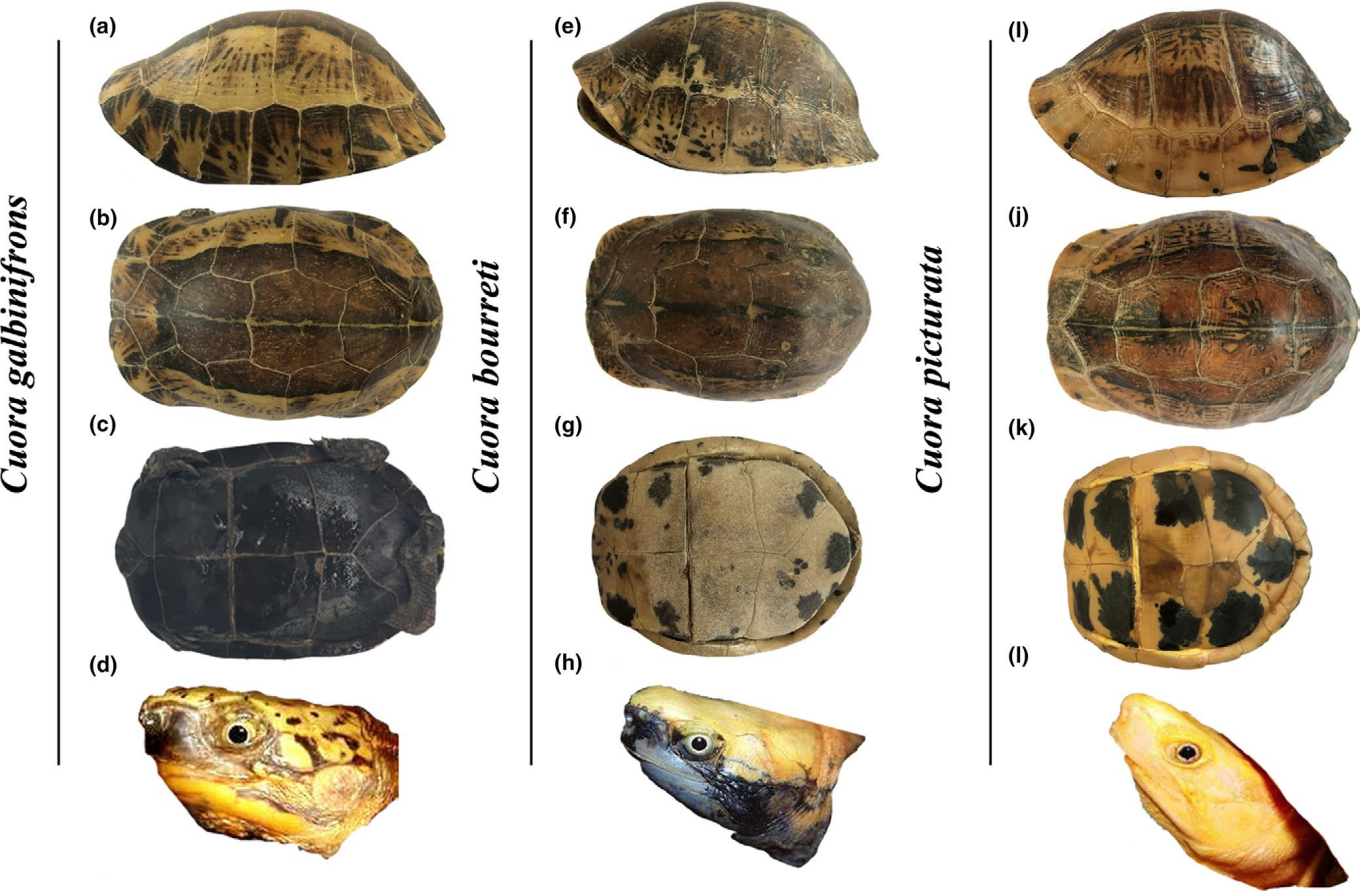
Shell morphology, head coloration, and patterning and coloration of plastron in *C*. *galbinifrons*, *bourreti*, and *picturata*

**Figure 2:**
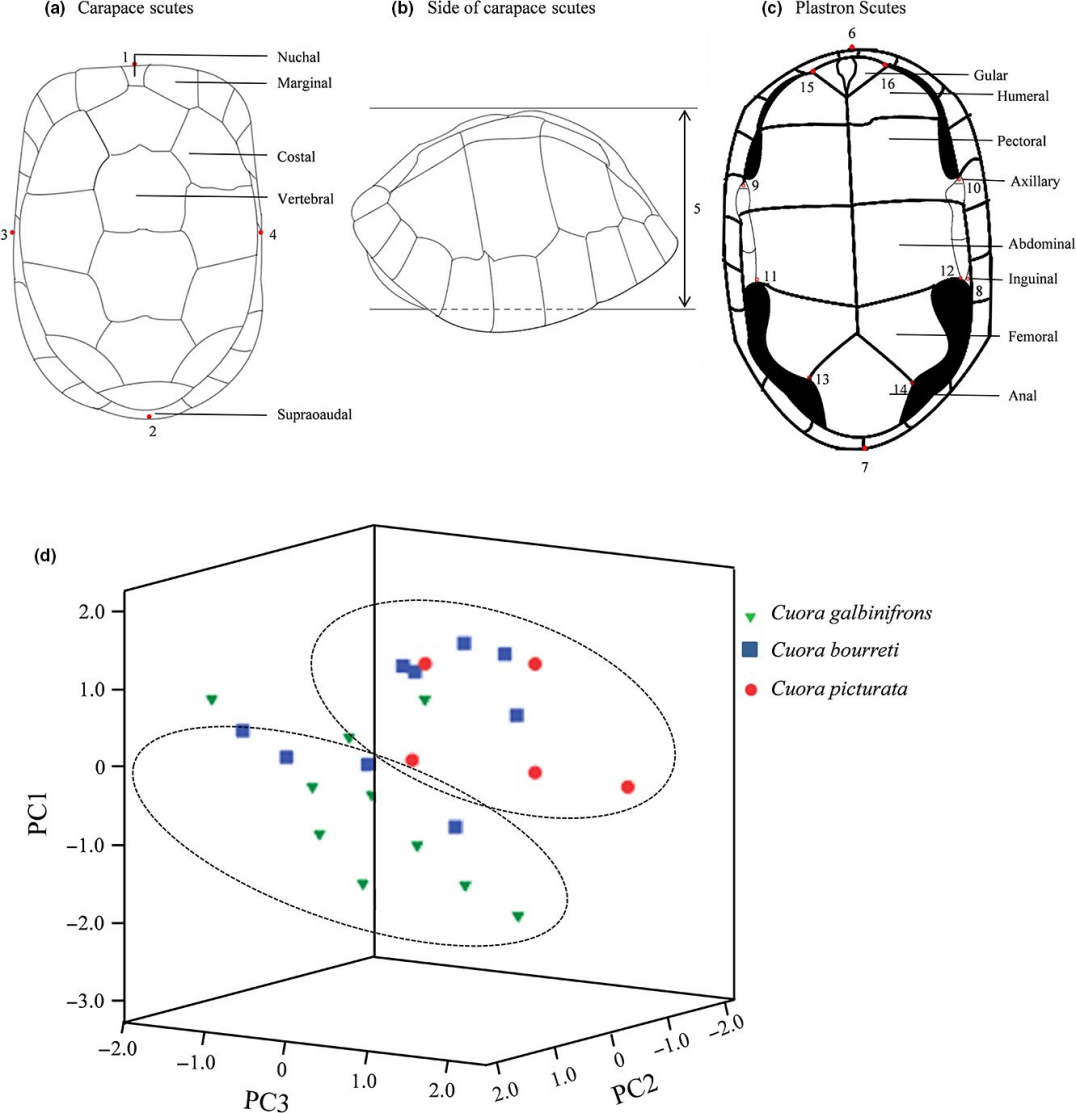
Morphological variation analysis among *C*. *galbinifrons*, *bourreti*, and *picturata*. (a–c) Locations of eleven homologous landmarks for morphological analyses of the three *Cuora* species. Detailed information on the 11 morphological characters is provided in Table 1. (d) Principal component analysis (PCA) based on 11 geometric morphometrics. The green triangle, blue box, and red circle represent *C*. *galbinifrons*, *bourreti*, and *picturata*, respectively

A total of 10 *galbinifrons* (eight specimens and one live turtle), nine *bourreti* (eight specimens and one live turtle), and five live *picturata* were obtained from Gaoming turtle breeding farm, Foshan city. Each specimen was photographed with a digital camera (Figure 1), and its nails were obtained and placed in absolute ethyl alcohol (Weijia Biotechnology Co., Ltd.) for one night and then transferred to −20°C conditions for group genetic analysis. Subsequently, we selected morphological landmarks to provide a precise definition of the turtle morphology. A total of 11 morphological characters were measured (Figure 2a–c and Table 1). Each morphological character was detected three different times to ensure its repeatability. We declare that the animal experimentation has been approved by the Pearl River Fisheries Research Institute (PRFRI, authorization number 45541566–7). All turtles used in this study were treated humanely and ethically, and we followed all applicable Chinese institutional animal care guidelines.

**Table 1:** Eleven morphological characters of the specimens

Code	Morphometric characters	Measurement
M1	Weight	–
M2	Carapace length	1–2 (Maximum linear length of carapace scutes)
M3	Carapace width	3–4 (Maximum linear width of carapace scutes)
M4	Body height	5 (Maximum linear height between carapace scutes and plastron scutes)
M5	Plastron length	6–7 (Maximum linear length of plastron scutes)
M6	Length of second half of the epigastrium	7–8 (Maximum vertical straight line length from inguinal shield to rear edge of tail shield)
M7	Anterior half width of abdominal armor	9–10 (Maximum linear width in front of axillary shield)
M8	Posterior half anterior width of abdominal armor	11–12 (Linear width at the junction of abdominal shield and femoral shield)
M9	Posterior half posterior width of abdominal armor	13–14 (Linear width at the junction of femoral shield and anal shield)
M10	Tortoiseshell bridge length	8–10 (Linear length from axillary shield to inguinal shield)
M11	Throat shield width	15–16 (Maximum linear width of gular shield)

### 2.2 Analysis of morphological differences

A homogeneity test of variances and one‐way analysis of variance (one‐way ANOVA) in SPSS version 20.0 were used to test for intergroup variation of the above 11 morphological characters. Duncan's multiple comparison was conducted on the variables with homogeneity of variance, and Tamhane's T2 method was used to analyze the variables without homogeneity of variance (significant level *p* = .05). Principal component analysis (PCA) was performed to highlight the differences of 11 morphological characters among the three *Cuora* species by SPSS version 20.0. The KMO and Bartlett's tests were used to detect whether the 11 morphological characters were suitable for factor analysis. All PCAs with eigenvalues >1.00 were considered important (Chatfield & Collins, 1983), and the first three principal components with cumulative contribution rates >85% were selected.

### 2.3 DNA extraction and nucleotide sequencing

Genomic DNA was extracted from the turtle nails by DNA Kit (Omega) following the manufacturer's protocol. Fifteen pairs of primers (Table S1) were designed to amplify the complete mitochondrial genome of *C. galbinifrons*, *bourreti*, and *picturata* according to previously published mitogenomes of the genus *Cuora* (Li et al., 2015). The PCR conditions were as follows: 95°C for 2 min, 35 cycles at 94°C for 30 s, 53–58°C for 30 s, 72°C for 2 min, and 72°C for 10 min, and the reaction system was performed in an Ex Taq (Takara) 40 μl reaction. The PCR products were sequenced and annotated by Shenggong Biotechnology. Subsequently, a pair of primers *COI*‐F and *COI*‐R (Table S1) was designed to amplify a partial fragment of the *COI* gene for 24 specimens. The total PCR volume was 25 μl containing 2.5 μl of 10× buffer (TaKaRa), 4 μl of dNTP (2.5 mM), 0.5 μl of each primer (10 μmol), 1 μl of template DNA, and 0.5 μl of Taq DNA polymerase (5 U/μl). The PCR conditions were as follows: 94°C for 2 min, 33 cycles at 94°C for 30 s, 55°C for 30 s, and 72°C for 2 min, followed by a final extension at 72°C for 10 min. The PCR products were sequenced by Shenggong Biotechnology. Moreover, we used the following primers (*Rag1*‐F, *Rag1*‐R, Table S1) for amplifying and sequencing partial fragments of the *recombination activating protein 1* (*Rag1*) gene. The reaction system was performed in an Ex Taq (Takara) 50 μl reaction mix, and the PCR condition were as follows: 94°C for 2 min, 94°C for 30 s, 58°C for 30 s, and 72°C for 2 min for 33 cycles. The positive products were purified with a by Gel Extraction Kit (Omega) and cloned into a PMD19‐T vector (Takara) for sequencing.

### 2.4 Sequence analysis

The data sets of nucleotide sequences were edited and assembled by Seqman software (Zhou et al., 2012). Sequence alignments and information on nucleotide variation were conducted by MEGA6.0 (Tamura, Stecher, Peterson, Filipski, & Kumar, 2013). Comparison of the *C. galbinifrons* complex with other available mitogenomes including *Cuora amboinensis*, *Cuora mouhotii*, *Cuora aurocapitata*, *Cuora cyclornata*, *Cuora pani*, *Cuora trifasciata*, and *Cuora flavomarginata* were made using the CGView Comparison Tool (CCT). For the *COI* and *Rag1* gene data sets, HKY+G and HKY were selected as the best fit models of evolution, respectively, by MODELTEST version 3.7 (Posada & Crandall, 1998). Maximum likelihood (ML) phylogenetic trees were implemented in MEGA6.0 (Tamura et al., 2013), and Bayesian inference (BI) trees were conducted in MRBAYES 3.1.2 (Ronquist & Huelsenbeck, 2003). For the BI tree, four simultaneous Metropolis‐coupled Monte Carlo Markov chains were used and run for 50,000,000 generations. Convergence to stationarity was evaluated using log‐likelihood values by TRACER v.1.5. The first 80% of the trees were discarded as burn‐in, and the remaining tree samples were used to generate a consensus tree. For the ML tree, the nodal support value was assessed from 100 nonparametric bootstrap replicates.

## 3 RESULTS

### 3.1 Morphological variation among the *C. galbinifrons* complex

A total of 24 individuals including 10 *galbinifrons*, nine *bourreti*, and five *picturata* were obtained from Gaoming turtle breeding farm, Foshan city, and their morphological differences were significant across these three *Cuora* species. As shown in Figure 1a,b, the carapace was slightly long and narrow with variations in patterns and colors. The plastron of *galbinifrons* was predominantly black with white growth lines on both sides (Figure 1c). On the yellow head of *galbinifrons*, black or brown spots were present (Figure 1d). *Bourreti* exhibited higher carapaces than *galbinifrons*, usually with three black lines distributed on the spine on both sides (Figure 1e,f). For *bourreti*, most of the plastron was yellow, and black patches scattered around the edge of the plastron covering not more than 75% of the scute to no spots at all (Figure 1g). The head of *bourreti* was similar to that of *galbinifrons* (Figure 1h). *Cuora picturata* clearly differed from *galbinifrons* and *bourreti*, with their significantly higher domed carapaces (Figure 1i,j) and golden head with reticulated grayish stripes (Figure 1l). In contrast to the plastron of *galbinifrons* (Figure 1c) and *bourreti* (Figure 1g), *picturata* usually has a spot size covering <75% to more than 40% of the scute (Figure 1k).

To further investigate the differences in morphometric shape among the *C. galbinifrons* complex, eleven morphological characters were measured and are shown in Figure 2a–c and Table 1. Based on one‐way ANOVA, morphological measurements of the plastron length (M5), anterior half width of abdominal armor (M7), posterior half anterior width of abdominal armor (M8), and throat shield width (M11) showed significant variations across the three *Cuora* species (Table S2). All 4 significantly different measurements of individuals from *picturata* revealed lower values than the measurements from individuals of *galbinifrons* and *bourreti* (Table S2). Individuals of *galbinifrons*, compared with *bourreti*, had lower values in the anterior half width of abdominal armor (M7) and posterior half anterior width of abdominal armor (M8) as well as higher values of plastron length (M5) and throat shield width (M11; Table S2). Moreover, based on principal component analysis (PCA), there was an overlap of specimens from different groups in which some individuals of *galbinifrons* and *bourreti* clustered into one group, and all *picturata* and some *galbinifrons* clustered into the other group (Figure 2d). A moderate level of interspecific difference among the *C. galbinifrons* complex was detected by three components in the PCA which explained 88.478% of the variation (Table 2). The first component, which explained 60.382% of the variation, suggested a difference in morphological traits, namely on M5, M7, and M8, while M11 was most representative for the second and the third components which explained 79.210% and 88.478% of the accumulated variation, respectively (Table 2).

**Table 2:** Factor loadings of principal components extracted from 17 proportional characters for *C*. *galbinifrons*, *bourreti*, and *picturata*

Morphometric characters	PC1	PC2	PC3
Characteristic root	6.642	2.071	1.019
Variance contribution (%)	60.382	18.828	9.268
Accumulative contribution (%)	60.382	79.210	88.478
Weight (g)	0.688	0.409	−0.364
Carapace length (cm)	0.943	0.107	−0.001
Carapace width (cm)	0.657	0.631	−0.237
Body height (cm)	0.442	0.778	−0.127
Plastron length (cm)	0.969	0.058	0.208
Length of second half of the epigastrium (cm)	0.852	−0.428	−0.243
Anterior half width of abdominal armor (cm)	0.946	−0.166	0.074
Posterior half anterior width of abdominal armor (cm)	0.760	−0.356	−0.190
Posterior half posterior width of abdominal armor (cm)	0.819	−0.447	−0.165
Tortoiseshell bridge length (cm)	0.749	−0.374	0.424
Throat shield width (cm)	0.530	0.456	0.681
ANOVA *p*‐value	.019	.172	.263

### 3.2 Nuclear DNA (nuDNA) phylogenies of *C. galbinifrons* complex

**Figure 3:**
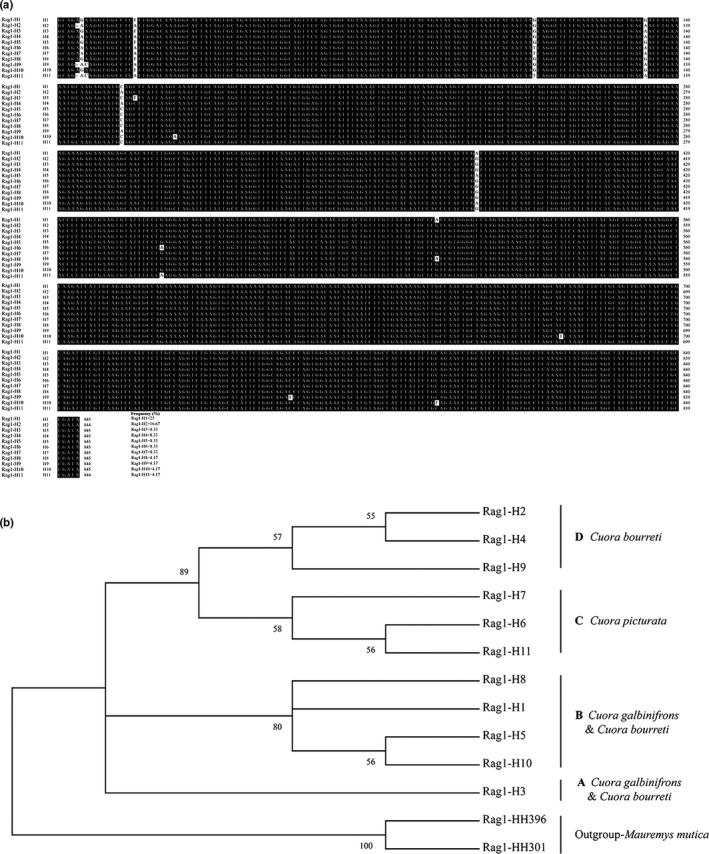
Multiple alignment and phylogenetic analysis of *Rag1* haplotypes across *C*. *galbinifrons*, *bourreti*, and *picturata*. (a) Multiple alignment of *Rag1* haplotypes in *galbinifrons*, *bourreti*, and *picturata*. The identical nucleotides are shown in black shadows. Gaps (−) are introduced to optimize identity analysis. The species names are shown to the left, and the occurrence frequencies are given at the end of the alignment. (b) ML phylogeny tree of *Rag1* haplotypes from *galbinifrons*, *bourreti*, and *picturata*. *Rag1* haplotypes of *Mauremys mutica* are used as out‐groups

The nuclear gene *Rag1* was used to reveal the phylogenetic relationship of the three *Cuora* species. A total of 11 *Rag1* haplotypes were detected from the three groups including 10 *galbinifrons*, nine *bourreti*, and five *picturata* (Figure 3a and Table S3). The length variations of 11 *Rag1* haplotypes ranged from 844 to 845 bp and contained 14 variable positions, of which nine were potentially parsimony informative. As shown in Figure 3a and Table S3, *Rag1‐H1* and *Rag1‐H2* were detected from the groups of *galbinifrons* and *bourreti*, respectively, and their total occurrence frequency was high, up to 41.67% (Figure 3a). Subsequently, the phylogenetic relationship generated using BI and ML from 11 *Rag1* haplotypes was well supported. As shown in Figure 3b, four major lineages emerged. Lineage A included only haplotype, *Rag1‐H3*, which was shared by *galbinifrons* and *bourreti*. Lineage B included four haplotypes, of which three haplotypes (*Rag1‐H1*, *Rag1‐H5*, and *Rag1‐H10*) were distributed in *galbinifrons* and 1 haplotype (*Rag1‐H8*) was from the *bourreti* group. Lineage C contained *Rag1‐H6*, *Rag1‐H7*, and *Rag1‐H11*, and all three haplotypes occurred in the group of *picturata* with occurrence frequencies of 8.33%, 8.33%, and 4.17%, respectively (Figure 3a,b). A total of three haplotypes (*Rag1‐H2*, *Rag1‐H4*, and *Rag1‐H9*) that were derived from the group of *bourreti* were clustered into lineage D.

### 3.3 Characteristics of the *C. galbinifrons* complex mitogenomes

In this study, the mitogenomes of the three *Cuora* species were sequenced and the assembled mitogenome sizes of *galbinifrons*, *bourreti*, and *picturata* were 17,200, 17,407, and 16,598 bp in length, respectively. As in typical vertebrates, all three *Cuora* species mitogenomes were composed of highly conserved genes including two ribosomal RNAs (rRNAs, 12S rRNA, and 16S rRNA), 22 transfer RNAs (tRNAs), 13 protein coding genes, and a variable control region which was also known as a displacement loop (D‐loop; Table 3). Among the 22 tRNAs, eight tRNAs (tRNA‐Gln, tRNA‐Ala, tRNA‐Asn, tRNA‐Cys, tRNA‐Tyr, tRNA‐Ser, tRNA‐Glu, tRNA‐Pro) were located on the heavy (H) strand, while the remaining genes were encoded by the light (L) strand except for *ND6* gene (Table 3). Twenty‐two tRNAs with lengths ranging from 67 to 78 bp were observed in these three *Cuora* species, and variable length polymorphisms of 22 intergenic spacers were detected from 1 to 26 bp (Table 3). Similar to most other vertebrates, ATG was the most frequent start codon for all the protein‐coding genes with only an exception in the *COI* gene, which had the GTG start codon (Table 3). For stop codons, six (*COII*, *ATP8*, *ATP6*, *ND4L*, *ND4*, *ND5*) of the 13 protein‐coding genes used TAA stop codons, three (*ND1*, *ND2*, and *ND3*) genes had TAG stop codons, two (*COI* and *ND6*) genes stop with AGG codons, and both *COIII* and *Cytb* genes were terminated through incomplete stop codon T (Table 3). The overall GC content of *galbinifrons*, *bourreti*, and *picturata* was 42.9%, 42.9%, and 42%, respectively. The control regions of these three *Cuora* species were highly divergent, and the length variations ranged from 1,087 to 1,886 bp.

**Table 3:** Details on the mitochondrial genome of *C*. *galbinifrons*, *bourreti*, and *picturata*

Gene/element	*Galbinifrons*/*bourreti*/*picturata*					
	Strand^a^	Size	GC percent (%)	Start codon	Stop codon	Intergenic nucleotide^b^
tRNA‐Phe	H/H/H	70/70/69	42.86/42.86/42.03	–	–	0/0/0
12S‐rRNA	H/H/H	965/965/963	40.73/40.83/41.95	–	–	0/0/0
tRNA‐Val	H/H/H	70/70/69	31.43/30.00/28.99	–	–	0/0/0
16S‐rRNA	H/H/H	1606/1605/1599	39.17/39.50/39.46	–	–	0/0/0
tRNA‐Leu	H/H/H	76/76/76	46.05/47.37/47.37	–	–	0/0/0
ND1	H/H/H	972/972/972	40.23/40.12/39.92	ATG/ATG/ATG	TAG/TAG/TAG	−1/−1/−1
tRNA‐Ile	H/H/H	70/70/70	48.57/47.14/47.14	–	–	−1/−1/−1
tRNA‐Gln	L/L/L	71/71/71	33.80/32.39/33.80	–	–	−1/−1/−1
tRNA‐Met	H/H/H	69/69/69	39.13/39.13/39.13	–	–	0/0/0
ND2	H/H/H	1041/1041/1041	40.15/40.25/39.19	ATG/ATG/ATG	TAG/TAG/TAG	−2/−2/−2
tRNA‐Trp	H/H/H	77/77/78	45.45/45.45/47.44	–	–	1/1/1
tRNA‐Ala	L/L/L	69/69/69	30.43/28.99/30.43	–	–	1/1/1
tRNA‐Asn	L/L/L	73/73/73	42.47/42.47/45.21	–	–	26/26/26
tRNA‐Cys	L/L/L	66/66/67	33.33/33.33/31.34	–	–	0/0/0
tRNA‐Tyr	L/L/L	71/71/71	40.85/40.85/40.85	–	–	1/1/1
COI	H/H/H	1548/1548/1548	42.05/41.99/41.73	GTG/GTG/GTG	AGG/AGG/AGG	−9/−9/−9
tRNA‐Ser	L/L/L	71/71/71	42.25/42.25/42.25	–	–	2/2/2
tRNA‐Asp	H/H/H	70/70/70	30.00/30.00/30.00	–	–	0/0/0
COII	H/H/H	687/687/687	37.41/37.70/38.28	ATG/ATG/ATG	TAA/TAA/TAA	2/2/2
tRNA‐Lys	H/H/H	73/73/73	43.84/43.84/43.84	–	–	1/1/1
ATP8	H/H/H	168/168/168	32.74/32.74/33.33	ATG/ATG/ATG	TAA/TAA/TAA	−22/−22/−22
ATP6	H/H/H	696/696/693	36.49/36.35/37.23	ATG/ATG/ATG	TAA/TAA/TAA	−1/−1/−1
COIII	H/H/H	784/784/784	41.96/41.58/42.60	ATG/ATG/ATG	T/T/T	0/0/0
tRNA‐Gly	H/H/H	68/68/68	29.41/27.94/32.35	–	–	0/0/0
ND3	H/H/H	351/351/351	36.47/36.75/37.32	ATG/ATG/ATG	TAG/TAG/TAG	−2/−2−2/
tRNA‐Arg	H/H/H	71/71/71	35.21/35.21/35.21	–	–	−1/−1/−1
ND4L	H/H/H	300/300/300	41.67/42.00/41.00	ATG/ATG/ATG	TAA/TAA/TAA	−7/−7/−7
ND4	H/H/H	1377/1377/1377	39.22/39.14/39.72	ATG/ATG/ATG	TAA/TAA/TAA	12/12/12
tRNA‐His	H/H/H	70/70/70	28.57/30.00/27.14	–	–	0/0/0
tRNA‐Ser	H/H/H	67/67/67	44.78/44.78/43.28	–	–	−1/−1/−1
tRNA‐Leu	H/H/H	72/72/72	38.89/38.89/38.89	–	–	0/0/0
ND5	H/H/H	1806/1806/1806	39.04/38.54/38.37	ATG/ATG/ATG	TAA/TAA/TAA	−5/−5/−5
ND6	L/L/L	525/525/525	39.43/39.24/38.48	ATG/ATG/ATG	AGG/AGG/AGG	0/0/0
tRNA‐Glu	L/L/L	68/68/68	42.65/42.65/38.24	–	–	4/4/4
Cytb	H/H/H	1144/1144/1144	43.44/43.36/42.83	ATG/ATG/ATG	T/T/T	0/0/0
tRNA‐Thr	H/H/H	72/72/72	37.50/37.50/34.72	–	–	1/1/1
tRNA‐Pro	L/L/L	69/69/70	42.03/40.58/35.71	–	–	0/0/0
D‐loop	H/H/H	1678/1886/1087	26.28/24.76/28.79	–	–	0/0/0

a H, heavy strand; L, light strand.

b Numbers indicate nucleotides separating two adjacent genes, while negative numbers indicate overlapping nucleotides.

### 3.4 Comparison with closely related turtle species

**Figure 4:**
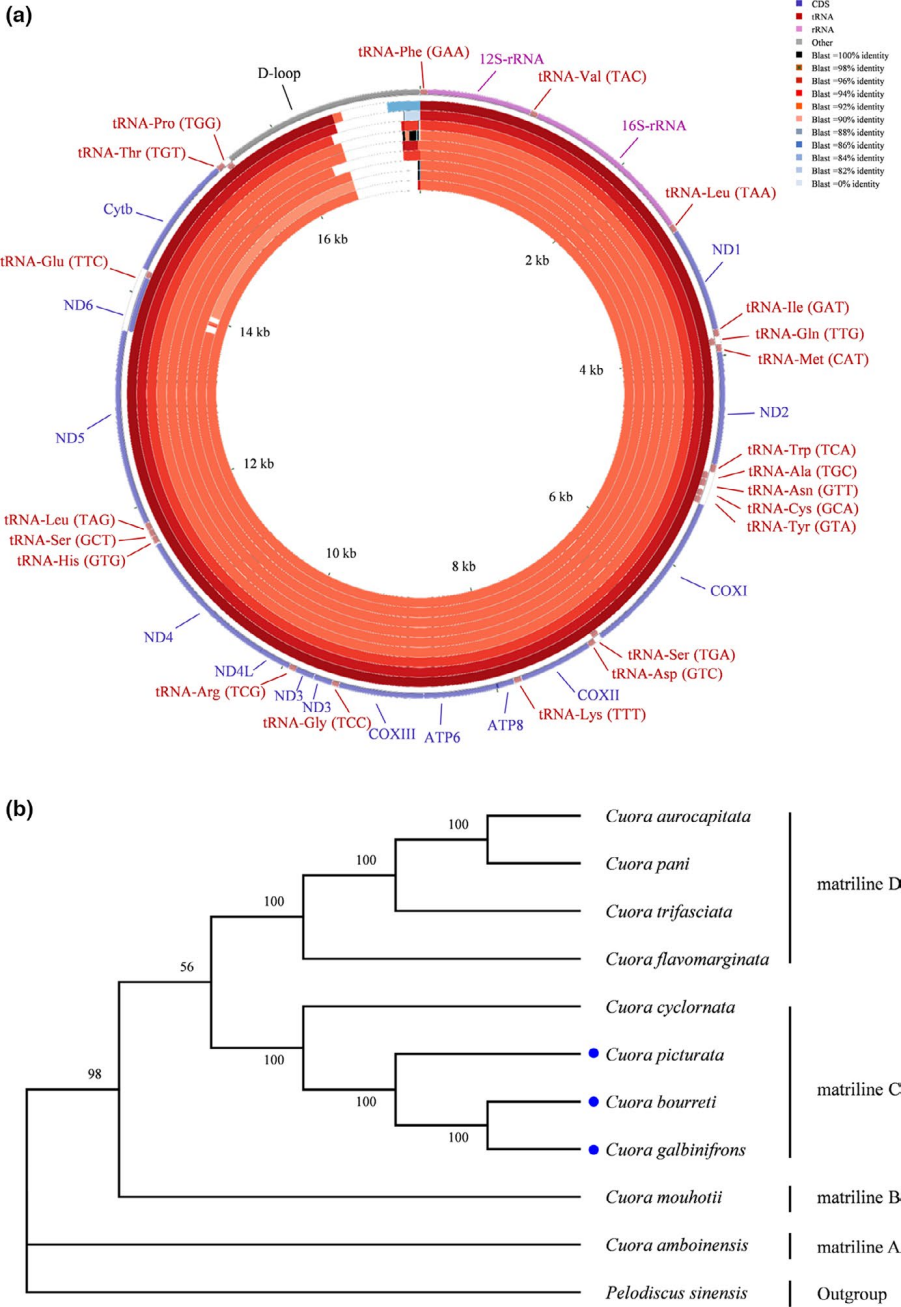
(a) Graphical map of the mitochondrial genome alignment results in nucleotide identity between *C*. *bourreti* and nine other turtle species based on the CGView comparison tool (CCT). Closeness to the outside of the circle indicates closer phylogeny to the reference sequence (*bourreti*). The rings labeled 1–9 represent alignment results of *C*. *bourreti* against *C*. *galbinifrons*, *C*. *picturata*, *C. mouhotii*, *C. flavomarginata*, *C. trifasciata*, *C. pani*, *C. aurocapitata*, *C*. *cyclornata*, and *C. amboinensis*. (b) ML phylogenetic tree of 10 *Cuora* species. The mitochondrial genome of *Pelodiscus sinensis* is used as an out‐group

Sequence alignment analysis of mitogenomes based on seven turtles species closely related to these three *Cuora* species including *Cuora amboinensis*, *Cuora mouhotii*, *Cuora aurocapitata*, *Cuora cyclornata*, *Cuora pani*, *Cuora trifasciata*, and *Cuora flavomarginata* in previous reports together with the current study revealed that the genome composition and gene arrangements were similar in all 10 turtle species that were compared (Figure 4a). Similar to the three *Cuora* species, the length and genetic variation were mainly located in the control region. The sequence identity at the nucleotide level between *bourreti* and the other turtle species varied between 83.47% and 96.11% (Figure 4a). To pursue the phylogenetic relationship of the 10 turtle species, both ML and BI trees were constructed according to their mitogenomes. Only the ML tree is shown in Figure 4b, as both BI and ML tree produced the same topology and well supported each other. Both matriline A and matriline B consisted of only one species (*C. amboinensis* and *C. mouhotii*, respectively). The sister group of matriline B and matriline C, contained *C*. *cyclornata* and the three *Cuora* species sampled in this study. Four species, *C. aurocapitata*, *C. pani*, *C. trifasciata*, and *C. flavomarginata* clustered into matriline D. The results from the topological trees of these species were consistent with the reported traditional phylogenies (Li et al., 2015; Spinks et al., 2012).

### 3.5 MtDNA phylogenies of the *C. galbinifrons* complex

**Figure 5:**
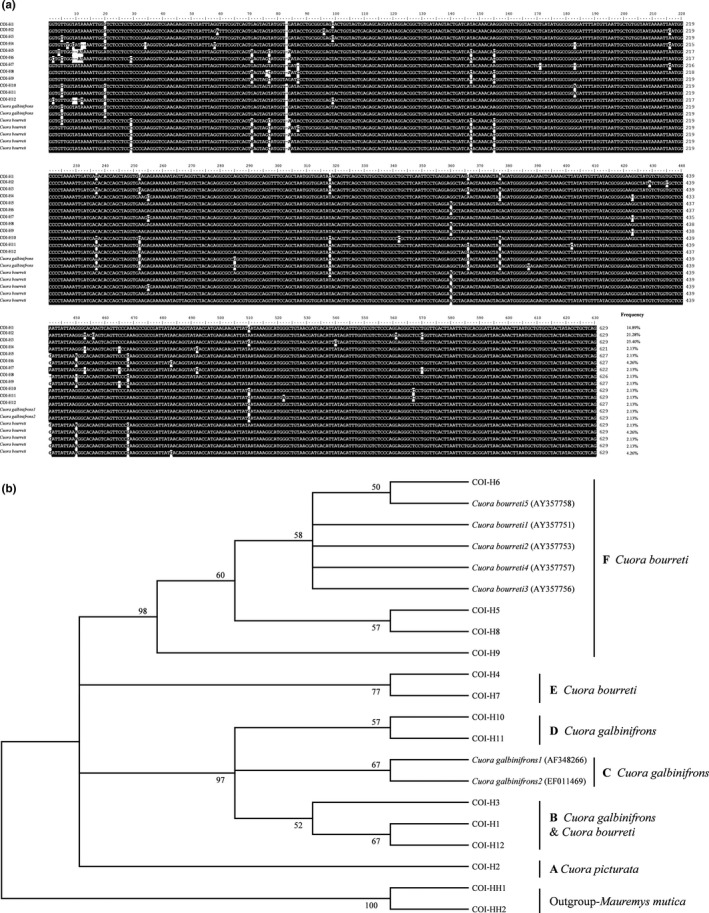
Multiple alignment and phylogenetic analysis of *COI* haplotypes across *C*. *galbinifrons*, *bourreti*, and *picturata*. (a) Multiple alignment of *COI* haplotypes in *C*. *galbinifrons*, *bourreti*, and *picturata*. The identical nucleotides are shown in black shadows. Gaps (−) are introduced to optimize identity analysis. The species names are shown to the left, and the occurrence frequencies are given at the end of the alignment. (b) ML phylogeny tree of *COI* haplotypes from *C*. *galbinifrons*, *bourreti*, and *picturata*. *COI* haplotypes of *Mauremys mutica* are used as out‐groups

In addition to nuclear gene *Rag1*, we generated sequence data for the partial *COI* gene to further investigate the genetic differentiation across the three *Cuora* species groups. As shown in Figure 5, a total of 19 mtDNA *COI* haplotypes including 10 haplotypes currently identified from samples in this study, seven haplotypes obtained from previous reports (Spinks & Shaffer, 2007; Stuart & Parham, 2004), and two shared haplotypes were identified from 21 *galbinifrons*, 16 *bourreti*, and 10 *picturata* (Figure 5a and Table S4). Variable length polymorphisms were also observed from 621 to 629 bp, and these 19 haplotypes included 47 variable positions, of which 30 were parsimony informative. Among the 19 haplotypes, the highest occurrence frequency, *COI‐H3* (23.40%) was only present in *galbinifrons* groups (Figure 5a and Table S4). *COI‐H2*, with a 21.28% occurrence frequency, originated directly from *picturata*. There was only one haplotype, *COI‐H1*, with a 14.89% occurrence frequency, which was shared by individuals from *galbinifrons* and *bourreti* groups (Figure 5a and Table S4). The remaining haplotypes with occurrence frequencies range from 2.13% to 4.26% were identified only in individuals of either *bourreti* or *galbinifrons* (Figure 5a and Table S4). The constructed BI and ML trees based on the mtDNA *COI* haplotypes resolved extremely similar matrilines and only the ML tree is shown in Figure 5b. The in‐group consisted of six highly supported matrilines. Matriline A, the sister group of all other matrilines, contained only one haplotype of *picturata*. Matriline B, the sister group of matrilines C, D, E, and F, included groups of *galbinifrons* and *bourreti* (Figure 5b). The remaining haplotypes of *galbinifrons* and *bourreti* were grouped in matrilines C and D and matrilines E and F, respectively (Figure 5b).

## 4 DISCUSSION

Inconsistencies between taxonomy and phylogenetic patterns have been revealed to be common among closely related species due to divergent markers, hybridization and introgression of wild populations, arbitrary sections of clines, artificial subdivisions of species or a random combination of these factors (Naksri, Tong, Lauprasert, Suteethorn, & Claude, 2013; Petit & Excoffier, 2009; Zhang, Nie, Huang, Pu, & Zhang, 2009; Zink & Barrowclough, 2008). Defining the interspecific relationships and species boundaries for *Cuora* has been a challenging endeavor for the systematic and conservation communities. Moreover, the concept of species and its criteria are widely controversial in previous literature (Meier, 2000). Here, we adopt the version of the phylogenetic species concept proposed by Crowe (1999), wherein trivial characters were avoided to define evolutionary units, and multiple independent lines of evidence including morphology, ecology, behavior, molecules, or physiology were investigated to determine species. In this study, morphological differences did not entirely separate the three species (Figure 1). Using PCA on 11 morphological characters of the *C. galbinifrons* complex, we found that *bourreti* partially overlapped in their morphological characters with the other two species (Figure 2). In addition, similar sets of relationships based on analyses of nuclear *Rag1* gene and mitochondrial *COI* gene indicated that *picturata* was well defined as a full species, but did not resolve *galbinifrons* and *bourreti* into two distinct groups (Figures 3 and 5). Our findings were consistent with osteological comparison of the *C. galbinifrons* complex (Fritz et al., 2006) but were slightly different from the hypothesis of Stuart and Parham (2004) which rendered *galbinifrons*, *bourreti*, and *picturata* as monophyletic, evolutionary lineages. Therefore, together with external morphology and molecular analyses, we recommend maintaining *picturata* as a full species, while *galbinifrons* and *bourreti* seem to be only subspecifically distinct. It is possible that species are gradually forming and will ultimately evolve into full species through the accumulation of variation but this is not evident yet.

Four possible explanations are suggested to explain the lack of remarkable divergence between *galbinifrons* and *bourreti*. First, as clarified by Fritz et al. (2006), *galbinifrons* and *bourreti* could be conspecific due to a consistent peculiar state in the bony carapace regarding the articulation of the rib tips with the peripheral plates, although differences in the shape of the shell and coloration of soft body parts were presented as attributable to polymorphism or geographic variation. Moreover, *bourreti* might have recently diverged from *galbinifrons*, and strong selection may have accelerated morphological differentiation while mutation rates of neutral genetic markers do not always reflect morphological differences in the process of phylogenetic evolution (Stuart & Parham, 2004). This lack of phylogenetic structure and limited lineage differentiation on mitochondrial DNA divergence are common among *Hemiculter leucisculus* from a lake complex in the middle Yangtze (Cheng et al., 2018) and a cluster of populations of the “Cumaná guppy” (*Poecilia reticulata* Peters; Alexander & Breden, 2010). In addition, as an integral part of the evolutionary process, hybridization and introgression were extensively found across a wide range of taxa in nature (Baldassarre, White, Jordan, & Webster, 2015; Kidd, Bowman, Lesbarrères, & Schulte‐Hostedde, 2010; Stuart & Parham, 2007; Twyford & Ennos, 2012). In terms of geographical distribution, *picturata* is currently only known from southern Phu Yen and Khanh Hoa provinces in southern central Vietnam (McCormack, Shi, & Stuart, 2016), and *galbinifrons* is found in North Central Vietnam, northeastern Laos, Hainan Island, and Guangxi Province of China (McCormack, Stuart, & Blanck, 2016), *bourreti* is distributed in northeastern Cambodia, eastern Laos, and central Vietnam south to northern Phu Yen Province (McCormack & Stuart, 2016) which revealed a partial intergradation area with that of *galbinifrons* (Stuart & Parham, 2004, Naksri et al., 2013). Therefore, it is reasonable to maintain *picturata* as a full species and assume *galbinifrons* and *bourreti* as subspecies of *C*. *galbinifrons* due to their coherent distribution area with no real boundaries. In addition, the overlap of distribution areas between *galbinifrons* and *bourreti* provided opportunities and possibilities for their hybridization, and hybrids from the same maternal species in captive or wild populations might generate highly homologous or identical mitochondrial DNA sequences. Finally, the relationship among closely related species might be obscured by variable morphological indices or molecular markers such as different nuclear and mitochondrial markers implemented in *Carassius* species complex (Liu, Jiang, et al., 2017; Luo et al., 2014), *Quasipaa boulengeri* (Yan et al., 2013), and *Neosalanx taihuensis* (Zhao et al., 2008). As a consequence, multiple molecular markers or genomic DNA of populations and larger sample sizes of known provenance will help to further investigate their taxonomic status.

The identification of the *C. galbinifrons* as full species with *C. g. bourreti* as its subspecies and *C. picturata* as a valid species plays an important role in the protection of their germplasm resources. The analysis of nuclear and mitochondrial DNA, in combination with morphology, led us to maintain *picturata* as full species and recommend *galbinifrons* and *bourreti* as subspecies of *C. galbinifrons*. Captive breeding efforts should be used to prevent genetic contamination and maintain the integrity of these phyletic evolutions by propagating them separately under artificial conditions. Moreover, China and Vietnam are the top two countries in need of effective turtle conservation in Asia (Stuart & Thorbjarnarson, 2003). Maintaining *picturata* as full species but downgrading *bourreti* as a subspecies of *C. galbinifrons* decreases the number of species in the *Cuora* genus, but will increase awareness of species‐level protection opportunities and conservation activities of *C. galbinifrons*. However, devastation of the *C. galbinifrons* complex from overexploitation for sale has not stopped, which makes further investigation of great significance across the spectrum of ecology, thremmatology, conservation, and evolution biology.

## CONFLICT OF INTEREST

The authors have no conflicts of interest to declare.

## AUTHOR CONTRIBUTIONS

Xiaoli Liu conducted the experiments and prepared the manuscript; Wei Li, Zhaoyang Ye analyzed the data; Xiaoyou Hong collected the samples; Xinping Zhu designed the study and wrote the manuscript. All authors read and approved the final manuscript.

## Data Availability

The data are provided in Supporting Information Appendix S1.
