# Supplementary material for: Morphological characterization and phylogenetic relationships of Indochinese box turtles—The Cuora galbinifrons complex
Source: Ecol Evol. 2019 Oct 2;9(23):13030–42. doi: 10.1002/ece3.5680 (PMC6912918; doi:10.1002/ece3.5680)
Supplement: Supplementary file 1 [file ECE3-9-13030-s001.docx]

**Supporting Information Appendix S1**

Table S1 Primers and their sequences used in this study

| primer | Sequence (5’-3’) | Experiment |
| --- | --- | --- |
| *Rag1*-F | TGGGAACAAGAGGCATAAAGAG | Amplification of *Rag1* gene |
| *Rag1*-R | AACAGGGTAGTACGGAGCAGGG |  |
| *COI*-F | CCTATATTTGATTTTCGGGGC | Amplification of *COI* gene |
| *COI*-R | CTTCGGGGTGACCAAAGAATCAAA |  |
| F1 | AAAGCATGGCACTGAAGTTGC | Amplification of mitochondrial genome |
| R1 | TTTCATCTTTCCCTTGCGGTAC |  |
| F2 | AAAGCATTCAGCTTACACCTGA |  |
| R2 | AAGTTCCACAGGGTCTTCTCG |  |
| F3 | TAATGCCTGCCCAGTGACA |  |
| R3 | TGATTCCGAGGGTTACTTC |  |
| F4 | TCAGGCTGAGCTTCAAACTC |  |
| R4 | GTAGTTGGGTTTGATTTAGCCC |  |
| F5 | ACCTGACAAAAACTAGCACCA |  |
| R5 | ACTATACCTGCTCAGGCCCCG |  |
| F6 | TACCTGTGTTTTTAACCCGTTGAT |  |
| R6 | TGGGTGAATCCTGCTATAA |  |
| F7 | GCCATCCCAACGGGAGTAAAAG |  |
| R7 | GCTATCCTGTTTAGCTTCTGTAG |  |
| F8 | GCCGAAGACGTCTTACACTCAT |  |
| R8 | GTTATAAGTAATGCTGCTGCTGC |  |
| F9 | GTCTTTATCTACAAGAAAAC |  |
| R9 | AAAAAATCGAATTGAGAATGG |  |
| F10 | AGTACAAGTGACTTCCAATTAC |  |
| R10 | TTTGGCCGCCTCAACGTGTA |  |
| F11 | GAACCAACTCCACGAAAACG |  |
| R11 | GCTATTTTTACAGTTGTTTTTG |  |
| F12 | AGGATAGAAGCAATCCACTGG |  |
| R12 | TATTTTTCGAATGTCTTGTTC |  |
| F13 | TATACACGCCTTCTTCAAAGC |  |
| R13 | CTAGTAGTGACCCGAAGTTTCAT |  |
| F14 | AACCACTGTTGTGTTCAACTA |  |
| R14 | CAGTTTCAGTAAGTCGGCAG |  |
| F15 | AGGCCTCTGGTTAATGTGTT |  |
| R15 | TTGGGCTGTCATGGTGTGCCT |  |

Table S2 Results of one-way ANOVA and ranges of meristic characters from *C. galbinifrons*, *bourreti* and *picturata*.

| Code | Morphological  index | *galbinifrons* | *bourreti* | *picturata* | *P-*value |
| --- | --- | --- | --- | --- | --- |
| M1 | Weight | 706.64 ± 187.23 | 744.7 ± 150.64 | 652.29 ± 178.96 | 0.596 |
| M2 | Carapace length | 16.88 ± 1.65 | 16.03 ± 1.20 | 16.86 ± 1.13 | 0.505 |
| M3 | Carapace width | 12.56 ± 0.95 | 11.49 ± 1.19 | 11.79 ± 0.58 | 0.663 |
| M4 | Body height | 93.03 ± 8.55 | 97.09 ± 7.66 | 94.14 ± 4.08 | 0.631 |
| M5 | Plastron length | 18.07 ± 1.87 | 18.05 ± 0.93 | 16.54 ± 0.84 | 0.049 |
| M6 | Length of second half of the epigastrium | 5.69 ± 0.73 | 5.44 ± 0.78 | 5.29 ± 0.58 | 0.494 |
| M7 | Anterior half width of abdominal armor | 10.28 ± 1.17 | 11.05 ± 0.95 | 9.57 ± 0.53 | 0.038 |
| M8 | Posterior half anterior width of abdominal armor | 5.25 ± 0.56 | 5.52 ± 0.34 | 4.71 ± 0.63 | 0.030 |
| M9 | Posterior half posterior width of abdominal armor | 4.28 ± 0.54 | 3.94 ± 0.37 | 3.88 ± 0.35 | 0.152 |
| M10 | Tortoise shell bridge length | 7.77 ± 1.09 | 7.37 ± 0.75 | 6.88 ± 0.37 | 0.107 |
| M11 | Throat shield width | 1.88 ± 0.25 | 1.79 ± 0.15 | 1.33 ± 0.22 | 0.000 |

Table S3 *RAG1* haplotypes detected in the *C. galbinifrons* complex

| haplotype | *galbinifrons* | *bourreti* | *picturata* |
| --- | --- | --- | --- |
| RAG1-H1 | 6 | - | - |
| RAG1-H2 | - | 4 | - |
| RAG1-H3 | 1 | 1 | - |
| RAG1-H4 | - | 2 | - |
| RAG1-H5 | 2 | - | - |
| RAG1-H6 | - | - | 2 |
| RAG1-H7 | - | - | 2 |
| RAG1-H8 | - | 1 | - |
| RAG1-H9 | - | 1 | - |
| RAG1-H10 | 1 | - | - |
| RAG1-H11 | - | - | 1 |

Table S4 *COI* haplotypes detected in the *C. galbinifrons* complex

| haplotype | *galbinifrons* | *bourreti* | *picturata* |
| --- | --- | --- | --- |
| COI-H1 | 5 | 2 | - |
| COI-H2 | - | - | 10 |
| COI-H3 | 11 | - | - |
| COI-H4 | - | 1 | - |
| COI-H5 | - | 1 | - |
| COI-H6 | - | 2 | - |
| COI-H7 | - | 1 | - |
| COI-H8 | - | 1 | - |
| COI-H9 | - | 1 | - |
| COI-H10 | 1 | - | - |
| COI-H11 | 1 | - | - |
| COI-H12 | 1 | - | - |
| *galbinifrons1* (AF348266) | 1 | - | - |
| *galbinifrons2* (EF011469) | 1 | - | - |
| *bourreti1* (AY357751) | - | 1 | - |
| *bourreti2* (AY357753) |  | 2 | - |
| *bourreti3* (AY357756) | - | 1 | - |
| *bourreti4* (AY357757) | - | 1 | - |
| *bourreti5* (AY357758) | - | 2 | - |
